# Effect of the phase of the season and contextual variables on match running performance in Spanish *LaLiga* football teams

**DOI:** 10.5114/biolsport.2024.133667

**Published:** 2024-04-08

**Authors:** Joaquín González-Rodenas, Jordi Ferrandis, Víctor Moreno-Pérez, Del Campo López, Ricardo Resta, Juan Del Coso

**Affiliations:** 1 Sport Sciences Research Centre, Rey Juan Carlos University, Fuenlabrada, Spain; 2 Sports Research Center, Miguel Hernandez University of Elche, Alicante, Spain; 3 Department of competitions and Mediacoach, LaLiga, Madrid, Spain

**Keywords:** Soccer, Match demands, Team sports, Motion analysis, Physical performance

## Abstract

This study aimed to examine the intra-season variation and the effects of contextual variables on total distance (TD), high-intensity running distance (HIRD) and high-speed distance (HSD) covered by Spanish football teams. The sample included 20 professional teams that competed in the Spanish LaLiga during the 2021–2022 season. Data were recorded using the TRACAB (ChyronHego, New York, USA) multicamera computerized optical tracking system. Multilevel linear mixed models were used to examine the effects of five contextual variables (1: phase of the season in six periods, 2: match location, 3: opponent ranking, 4: team ranking and 5: congested schedule) on TD, HIRD and HSD. Teams increased the running performance from period 1 to 5 of the season in TD (+2.2%; P < 0.05) and from period 1 to 4 in HIRD (+7.2%; P < 0.05), while a declining effect was found in period 6 for both TD (-1.2%; P < 0.05) and HIRD (-5.8%; P < 0.05). More HIRD (+1.5) and HSD (+5.3) were covered at home (P < 0.05), and more TD and HIRD were covered when playing against opponents ranked in the first (+2.4% and +5.3%, respectively; P < 0.05) and second quartile (+1.2% and +5.0%, respectively; P < 0.05), compared to teams from the fourth quartile. Football coaches and fitness specialists should note that running performance of Spanish teams increased throughout the season, but it declined during the last matches of the competition. Additionally, home matches and highranked opponents elicited higher running demands.

## INTRODUCTION

Professional football (soccer) is a physically demanding team sport characterized by the repetition of high-intensity activities such as accelerations, decelerations, changes of directions, sprinting, jumping, and dribbling over 90 min [1, 2]. As a consequence of these high physical demands during football match play, football players incur several signs of acute fatigue such as declining muscle strength [3], reductions in the sprinting and jumping capacities [4] and biochemical alterations (muscle damage, inflammatory and immunological markers, etc.) [5]. The acute signs of fatigue induced by a football match can persist for up to 72 hours or even longer after the match [6]. Moreover, the physical demands of professional football have considerably increased over the last decades in the main European competitions, especially in relation to the distance covered at high intensity and sprint [7, 8].

This heightened physicality of modern football makes it necessary for professional players to be highly trained to cope with the physical demands of the game, as well as to recover as quickly as possible between matches. In recent decades, professional football has experienced a progressive increase in the density of matches [9] and elite football teams can play between 50 and 60 matches per year in national and international competitions. To be successful, professional football squads require an optimal physical condition that must be preserved through the whole season [10]. However, with a competitive season lasting 9–10 months and teams frequently playing more than one match per week, chronic fatigue can be experienced across the season, ultimately affecting the distance covered per match during specific periods of the season [11]. Interestingly, scientific evidence has not consistently demonstrated a significant decrease in running performance during congested competitive periods [12, 13]. Nonetheless, recent studies have reported significant variations in physical performance throughout different phases of the season [14–16], suggesting a link between the chronic fatigue developed across the whole season and reduced match performance.

In early studies [17, 18] conducted in the Italian Serie A, variables such as total distance (TD) and high-intensity running distance (HIRD) increased towards the end of the season. In contrast, recent studies conducted in the Spanish [11] and German national leagues [14] found that TD and HIRD peaked at around two-thirds of the season but declined in the final matchdays of the season. Meanwhile, Springham et al. [16] observed large reductions in sprint performance, HIRD and TD across the season in an English Championship team. Surprisingly, Li et al. [15] observed a completely different trend in the Chinese Super League, where teams showed a ‘U’-shaped pattern with running performance reaching its highest level early in the season, gradually declining to its lowest point at two-thirds of the season, and progressively rebounding towards the end. So, it seems that there is no consensus to ascertain whether there is an effect of the accumulation of matches through the season on match running metrics. Perhaps the characteristics (team’s level, number of matchdays, congestion) of each national football league [19] and the introduction of the five-substitution option [20] are responsible in part for the lack of agreement among investigations. In any case, the study of the intra-season variation in different match running metrics with the actual conditions of professional performance deserves further investigation.

Additionally, in the analysis of match running performance across the season it is imperative to include the combined effects of contextual variables, as it has been empirically demonstrated that factors such as the venue of the match, the ranking of the opponent, and the ranking of the team itself exert considerable influence on match running performance [21]. In this regard, existing studies in different competitions have consistently revealed that home teams exhibited superior levels of running performance in comparison to the teams playing away [22–24]. Furthermore, playing against stronger opponents appears to elevate the physical demands of the teams [17, 25]. Conversely, the association between a team’s ranking and physical performance remains a subject of contention, as unrelated findings have been reported across different competitions [26–28], which suggests that match running performance does not guarantee greater success in professional football.

Therefore, the aim of this study was to examine the intra-season variation and the effects of contextual variables such as match location, own team’s level, opponent’s level and match schedule on total distance (TD) high-intensity running distance (HIRD) and highspeed distance (HSD) in teams competing in the Spanish *LaLiga* across the season. We hypothesized that football teams would experience a decline in all running metrics in the last matchdays of the season, while they would run more when playing at home and against high-ranked opponents.

## >MATERIALS AND METHODS

### Sample

The sample included 20 professional male football teams that competed in the first division of Spanish football (*LaLiga*) during the season 2021–2022, including the analysis of 760 team observations during 380 official matches. The collective and total running performance of each team during each match was registered. The competition started on 13 August, 2021 and finished on 22 May, 2022, lasting a total of 9 months and 9 days. The dataset utilized in this investigation was provided by *LaLiga*, which granted authorization for the analysis of pertinent variables and the dissemination of results for scientific purposes. In strict adherence to the ethical guidelines outlined by *LaLiga*, the present investigation does not contain any information that could lead to the identification of individual football players. Furthermore, all procedures and protocols employed in this study were conducted in accordance with the principles set forth in the Declaration of Helsinki and the Institutional Review Board of the Rey Juan Carlos University approved this study.

### Procedures

The design of this current descriptive and comparative investigation is based on suggestions made by Mackenzie and Cushion [29]. In this regard, this study takes multiple factors into consideration, including the sample (i.e., 20 elite football teams), competitive nature of football (i.e., official matches played over one season of a national football league), and the operational definitions of the investigated variables (i.e., match running metrics and contextual variables). Running data during the matches were captured using the TRACAB (ChyronHego, New York, USA) multicamera computerized optical tracking system, which has a sampling frequency of 25 Hz, and processed using the Mediacoach software (*LaLiga*, Madrid, Spain), which is a valid and reliable system to analyse football performance [30]. Specifically, for evaluating the match running performance of teams on each matchday, three independent variables based on total distance and high-speed distance [1] were analysed:

1. Total distance (TD): the number of metres that the team covered during the match.
2. High-intensity running distance (HIRD): the number of metres that the team covered during the match between 21 km · h^−1^ and 24 km · h^−1^
3. High-speed distance (HSD): the number of metres that the team covered during the match at > 24 km · h^−1^.

The data of this tracking system have been used in recent prestigious investigations that included the analysis of running performance in professional football teams [31, 32].

The independent variables of this study included five contextual variables:

1. Phase of the season: The season was divided into six different periods (Period 1: matchday 1–7; Period 2: matchday 8–13; Period 3: matchday 14–19; Period 4: matchday 20–25; Period 5: matchday 26–31; Period 6: matchday 32–38), based on a previous study with a similar objective [14].
2. Mach location: Home matches versus away matches.
3. Opponent’s ranking: The ranking of the opponent at the end of the season divided into four quartiles (First quartile: first to fifth position; Second quartile: sixth to tenth position; Third quartile: eleventh to fifteenth position; Fourth quartile: sixteenth to twentieth position).
4. Own team’s ranking: The ranking of the team at the end of the season divided into four quartiles (First quartile: first to fifth position; Second quartile: sixth to tenth position; Third quartile: eleventh to fifteenth position; Fourth quartile: sixteenth to twentieth position).
5. Congested schedule: Number of matches played in the same week [12, 13, 33] (1: non-congested matches: The team plays one match in the same week; 2: The team plays two matches in the same week).

### Statistical analysis

The data were transferred from Mediacoach to a .csv database which was organized in Microsoft Excel. All statistical analyses were carried out using the software IBM SPSS Statistics Version 27.0. Due to the hierarchical structure of teams’ performance in football (each team has its own tactical style), a multilevel mixed model [34] was performed to cluster the collective performance (level 2) into teams (level 1). With this organization of the data, a generalized mixed linear model was carried out to explore the effects of the contextual variables (fixed effects) on the running performance, considering the effect of the team (random effects). Thus, the “team effects” represented unobserved team characteristics that influence the running performance and account for the non-independence of the data [32]. Graphic charts with the predicted means and confidence intervals were displayed to show running performance of teams according to the contextual variables evaluated. Pairwise comparisons of the estimated means were performed through Fisher’s least significant difference test. The significance level was set to P < 0.050.

## RESULTS

Table 1 shows the coefficients and statistical differences concerning the effects of the phase of the season and other contextual variables on the match running demands. Regarding the effect of the phase of the season, a significant effect was found for TD, HIRD and HSD. Specifically, the TD covered showed an increasing effect as the season was progressing, reaching its largest increase in the fifth period of the season (Coeff = 2441.22; SE = 469.97; P < 0.001), in comparison with the first period of the season. For HIRD, an increasing effect was observed in comparison with the first period of the season, reaching its largest increase in the fourth period (Coeff = 267.28; ES = 55.86; P < 0.001). After this period, both TD and HIRD showed a decrease in the sixth period. For HSD, there was an increasing trend from the first to fourth period of the season, with a statistically significant difference between the first and the fourth period, when the HSD was higher (Coeff = 134.49; SE =55.86; P = 0.016).

**TABLE 1:** Comparative analyses of match running demands according to the phase of the season and other contextual variables.

	TD (m)		HIRD (m)		HSD (m)	

	Coeff (SE)	P	Coeff (SE)	P	Coeff (SE)	P
Intercept	114991 (991.75)	< 0.001	3806.31 (110.16)	< 0.001	2988.05 (99.02)	< 0.001

**Season phase**						
1: 1–7 matches						
2: 8–13 matches	930.10 (451.40)	**0.049**	70.17 (49.63)	0.158	15.41 (56.00)	0.783
3: 14–19 matches	1862.37 (468.67)	**< 0.001**	234.04 (49.23)	**< 0.001**	73.58 (55.54)	0.186
4: 20–25 matches	2412.88 (471.36)	**< 0.001**	267.28 (49.51)	**< 0.001**	134.49 (55.86)	**0.016**
5: 26–31 matches	2441.22 (469.97)	**< 0.001**	231.42 (49.36)	**< 0.001**	90.41 (55.69)	0.105
6: 32–38 matches	983.22 (451.40)	**0.030**	34.97 (47.41)	0.461	56.04 (53.49)	0.295

**Match location**						
Home						
Away	-215.03 (273.46)	0.432	-57.79 (28.72)	**0.045**	-156.18 (32.40)	**< 0.001**

**Opponent ranking**						
First quartile						
Second quartile	-1343.03 (387.44)	**0.001**	-11.99 (40.70)	0.768	112.71 (45.91)	**0.014**
Third quartile	-1825.11 (385.97)	**< 0.001**	151.05 (40.54)	**< 0.001**	-46.48 (45.74)	0.310
Fourth quartile	-2663.63 (386.06)	**< 0.001**	-197.57 (40.55)	**< 0.001**	-10.19 (45.75)	0.824

**Team ranking**						
First quartile						
Second quartile	540.08 (1266.42)	0.670	193.46 (142.34)	0.175	207.77 (120.46)	0.085
Third quartile	-86.727 (1268.23)	0.945	116.08 (142.52)	0.416	111.89 (120.73)	0.354
Fourth quartile	-1613.10 (1268.56)	0.204	-181.08 (142.55)	0.204	-118.553 (120.78)	0.327

**Congested match**						
Congested						
Not congested	-517.32 (296.07)	0.081	-46.38 (31.10)	0.136	-39.238 (35.06)	0.264

**FIG. 1:**
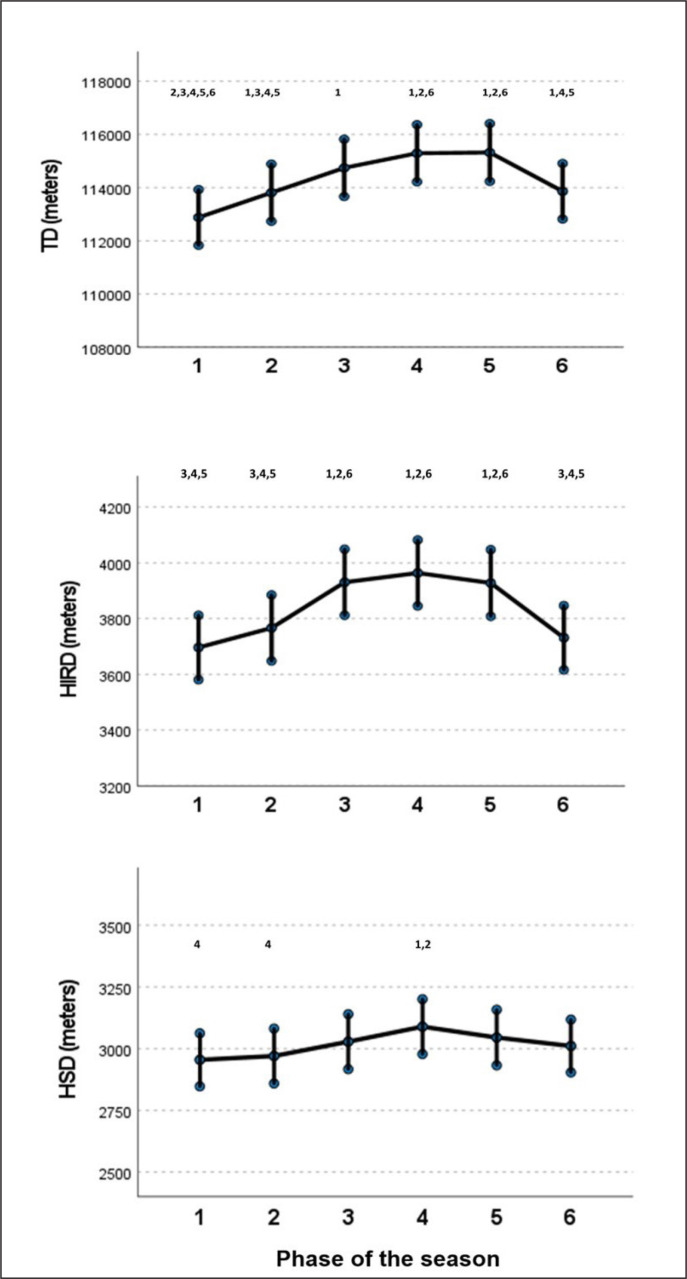
Estimated means and confidence intervals of TD, HIRD and HSD according to the phase of the season. Data correspond to the team’s running performance, including data of 11 players per team. 1 = different from 1; 2 = different from 2; 3 = different from 3; 4 = different from 4; 5 = different from 5; 6 = different from 6. Statistical differences = P < 0.05.

Concerning match location, significant decreases of HIRD (P < 0.001) and HSD (P < 0.001) were observed in away matches with regard to home matches, while no differences were found in TD (Table 2). As for the ranking of the opponent team, significant effects were observed for TD, HIRD and HSD (all P < 0.050). Particularly, a decreasing effect was observed in TD and HIRD as the ranking of the opponent was lower (Figure 1). For HSD, a significant difference was found between the first and second quartile (Coeff = 112.71; SE = 32.40; P = 0.014). Finally, no differences in match running performance variables were found for own’s team ranking or match schedule (Table 1).

Figure 1 shows the longitudinal evolution of TD, HIRD and HSD through the different periods of the season. For TD, an increasing effect was found from the first period (112877 m; 95% CI: 111829.75–113925-05) to the fifth period (+2.2%), while a decline of 1.2% was observed between the fifth and the sixth period of the season. As regards HIRD, an increasing effect was also found from the first period (3696.18 m; 95% CI: 3580.65–3811.71) to the fourth period (+7.2%) (P < 0.05) and a descending effect was observed between the fourth and the sixth period (-5.8%) (P < 0.05). Finally, a significant increasing difference was observed between the first and the fourth period regarding HSD (+4.5%) (P < 0.05), while no differences were found between the rest of the periods.

**FIG. 2:**
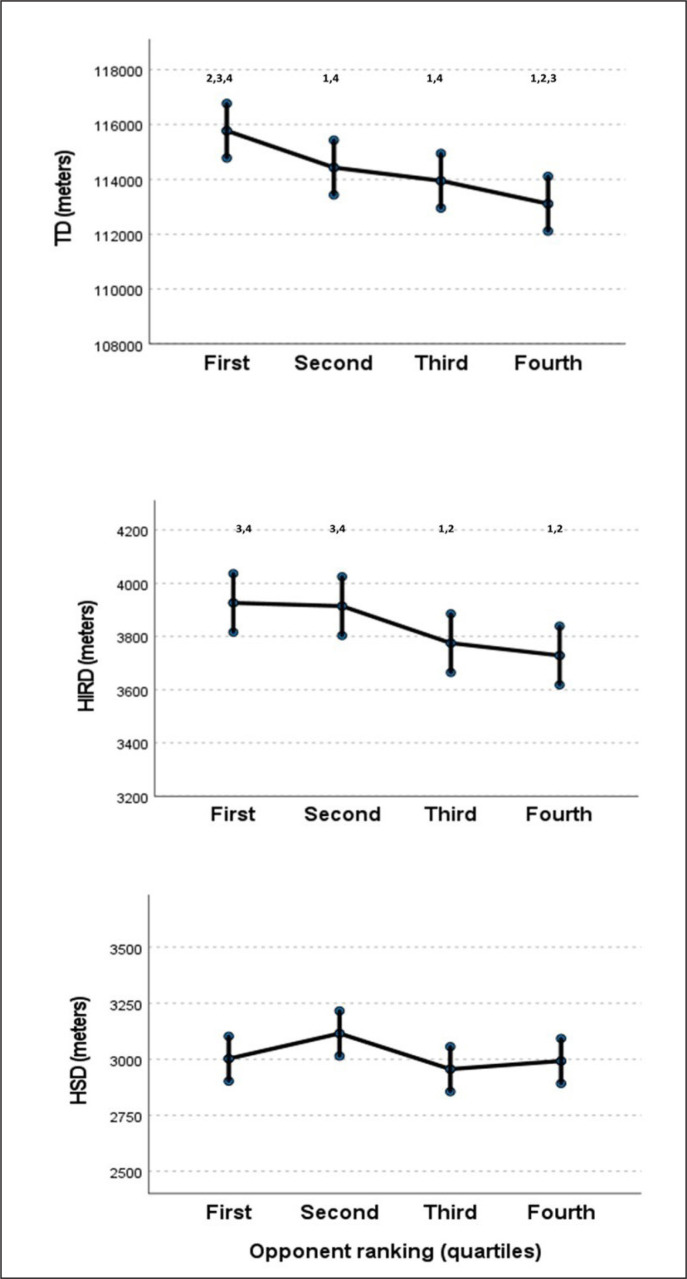
Estimated means and confidence intervals of TD, HIRD and HSD according to the opponent ranking in quartiles. Data correspond to the team’s running performance, including data of 11 players per team. 1 = different from 1; 2 = different from 2; 3 = different from 3; 4 = different from 4; 5 = different from 5; 6 = different from 6. Statistical differences = P < 0.05.

Figure 2 depicts the effects of the opponent ranking on TD, HIRD and HSD. For TD, the higher the ranking of the opponent was, the greater was the TD was covered by football teams. Specifically, playing against an opponent ranked in the first quartile demanded 2.3% more of TD, in comparison with the fourth quartile (P < 0.05). As regards HIRD, playing against teams ranked in the first or second quartile demanded more HIRD than playing against teams ranked in the third (+4.0%; +3.7%) and fourth quartile (+5.3%; 5.0%), respectively (P < 0.05). Finally, the opponents ranked in the second quartile were the teams that demanded more HSD, in comparison with the first (+3.8%), third (+5.4%) and fourth (+4.1%) (P < 0.05).

## DISCUSSION

The aim of this study was to examine the intra-season variation and the influence of several contextual variables on TD, HIRD and HSD in teams participating in the Spanish *LaLiga* in the 2021–2022 season. The analyses employed in this study included the assessment of a generalized linear mixed model, revealing significant effects of variables such as the phase of the season, opponent’s ranking and match location on all match running performance variables. Conversely, variables such as team ranking and congested match schedules did not exhibit any discernible impact on the running output of the teams. Collectively, the outcomes of this study are innovative because they describe how the running metrics during official matches of a national league vary across the season with peak values between match days 20–30 of a 38-matchday competition. Additionally, this analysis confirms that professional football teams adapt to the characteristics of each game, running more when they play at home and against top-tier rivals.

The findings of the current study demonstrated that match running performance (especially TD and HIRD) presented an inverted U-shaped trajectory. Teams displayed lower running performance during the initial phases of the season (periods 1 and 2) but exhibited a significant improvement in subsequent phases, maintaining their performance until the latter stages of the season (period 6), when values declined. Similar trends have been observed in previous investigations conducted in Spain [11, 35] and Germany [14], emphasizing the lower running performance observed at the beginning and end of the competitive season, compared to the middle periods. It is interesting to note that teams covered less TD, HIRD and HSD during the first period of the season, following the preseason period. Although previous studies have observed a fitness improvement of players after the preseason period [36–38], our research suggested that teams did not reach their peak match running performance until the middle phases of the season, when at least 12 matches had been played. This delay may be attributed to the players and teams adapting to the demands of real competition, which require higher physical capabilities than those encountered during friendly matches and the progressive training and playing load during preseason. Additionally, some studies have reported a decline in sprint time and repeated sprint ability following the preseason period [37, 39], possibly due to the high density of physical load experienced within this specific training period of the season. In practical terms, the inverted U-shaped trajectory for all running metrics presented in this investigation might be useful for strength and physical conditioning coaches as it reflects that the current activities performed in the transition period do not fully prepare players to attain maximal performance on the first matchdays of the season as they need more than 20 matchdays to obtain peak running performance. These outcomes also encourage professional teams to achieve high running performance from the first match after resuming the competition as it may be a competitive advantage with respect to the remaining teams. This has especial relevance for national leagues, in which the benefits (i.e., points earned) are the same in all matches of the season, but should not be applied for other football competitions such as knockout competitions (e.g., UEFA Champions League or World Cups), where a progressive tapering in running metrics towards the end of the competition may be beneficial.

On the other hand, a decline in match running performance was observed in the last period of the season, especially for TD and HIRD. Existing literature suggests that players experience increased high stress, fatigue [40], elevated levels of muscle damage biomarkers [41] and a higher incidence of injuries [42] towards the end of the season. These findings highlight the complex nature of the last phases of the season of professional football, wherein teams engage in crucial matches to achieve their competitive objectives while attempting to maintain their technical and tactical success without compromising their running performance. In modern football, which entails 50–60 matches per year in a combination of national and international competitions, successful professional football squads may need to enhance their physical conditioning to preserve a more stable running performance during the last matchdays of the season.

It is worth noting that other studies have observed different patterns of match running performance over the season in different competitions and specific teams. For instance, a recent investigation of Li et al. [15] found that Chinese teams showed U-shaped variation through the season, where the lowest match running performance was observed in the middle phases of the season, while the first and last parts of the season showed the highest performance. Differently, Springham et al. [16] evaluated a team competing in the English Championship and reported a decreasing trend in match physical performance throughout the season, while Smpokos et al. [43] observed lower physical performance during the second quarter of the season (November) and higher average levels during the fourth quarter of the season (February-March) in a Greek professional team. These divergent findings emphasize that while our study in Spanish football identified a specific trend in match running performance over the season, such trends can vary significantly depending on the context and the specific teams under investigation. So, until further investigations are available to understand how the characteristics of each national league influence the match running performance across the season, the current findings should be applied to football teams competing in *LaLiga* or national competitions with similar duration in matchdays and playing level.

Regarding the influence of the remaining contextual variables on running performance variables, our study revealed that Spanish teams demonstrated higher HIRD and HSD in home matches than when they played away. In line with this finding, a wide quantity of previous studies also observed higher physical demands in home matches [22–24, 44], although variations can be observed when examining specific teams [45, 46]. In this regard, home teams typically have greater winning expectations and a more offensive strategy than away teams [47], as well as displaying faster attacks [48, 49], and achieve greater offensive production [50]. Moreover, additional factors such as crowd effects, familiarity with the field, travel effects or psychological factors can also contribute to higher physical output during home games [51].

Playing against a higher-ranked opponent was also found to elicit greater physical demands, irrespective of the team’s own ranking, which had no effect on match running performance. Specifically, Spanish football teams ran greater distances when they played against topfive ranked teams, suggesting that these rivals posed higher demands on the football teams, probably because they have better technical and tactical characteristics that require the opponent team to run more to compensate potential deficiencies [52]. This finding aligns with previous studies conducted in diverse contexts [17, 22, 25]. In this regard, playing against high-ranked opponents seems to make it more difficult to sustain long attacks [48], which also entails spending more time without having the ball, as well as trying to be more vertical offensively [49]. This tactical context induced by the higherranked teams requires greater physical effort to neutralize the offensive production of the opposing team, which can explain the higher values in TD, HIRD and HSD. In fact, the study of Folgado et al. [25] reported that playing against stronger opponents implied that teams spent more time in synchronized behaviour and performing faster movements. In contrast, our study did not observe significant differences in running performance based on the team’s own ranking, which aligns with the conclusions drawn by Asian Clemente et al. [28] in their investigation of the Spanish *LaLiga*. This fact indicates that the final success of teams is not directly associated with higher match running performance, so other tactical and technical variables seem to be more important. Nevertheless, noteworthy observations from recent studies conducted on the Spanish *LaLiga* [53]and the German *Bundesliga* [26, 27] observed that high-ranked teams covered greater distances with ball possession and performed higher numbers of maximal velocity runs. This suggests the necessity of incorporating additional context-specific physical variables to explore potential discrepancies among teams occupying distinct ranking positions. Additionally, it seems that the best football teams cover the same running distance as lower-ranked teams, but they may differ in the distance running with the ball.

Lastly, no significant effects were observed on match running performance when teams played in congested schedules. Previous studies have indicated that players experience muscle damage up to 72 hours after a match [6] and face a higher risk of injuries during congested periods [33]. However, consistent with our results, scientific evidence has not demonstrated significant declines in running performance when competing in congested periods in professional football [13]. This suggests that despite the potential physiological and mental fatigue suffered by players during congested periods [54], their match running performance generally remains unaffected. In this regard, it is worth exploring new physical metrics to fully understand the real impact of these intensified competitive periods within the season [12]. For instance, a recent study by Djaoui et al. [55] revealed a decline in acceleration and deceleration performance during congested periods compared to non-congested ones. Therefore, analysing match running performance alone may not capture the true extent of congested periods’ impact on teams’ and players’ physical performance.

It is important to acknowledge the limitations of this study. Firstly, this investigation evaluated the match running performance of teams without considering the individual characteristics of players or specific playing positions. Secondly, the study evaluated one single season, which may reflect the specific conditions and team’s performance in that period. Thirdly, the current investigation was conducted exclusively with data from a national professional male football league in Spain, and caution should be exercised when generalizing the result to other competitions, categories, or women’s football.

## CONCLUSIONS

In conclusion, our study revealed an inverted U-shaped pattern in the match running performance of Spanish professional teams through the season. Specifically, running values such as TD, HIRD and HSD were lower at the outset of the season, showed a gradual increase leading to sustained performance, and subsequently declined in the final matches of the season. Additionally, match running performance was found to be higher when playing at home and when facing highranked opponents, whereas no significant effects were observed for variables such as team ranking and congested matches.

Regard to practical applications, our study holds considerable relevance for fitness coaches and performance directors of football clubs in comprehending seasonal variations and the influence of contextual variables on the match running performance of teams. Based on our findings, fitness coaches should design a suitable yearly training and recovery periodization with the objective of sustaining the match running performance of teams until the culmination of the competitive season and avoiding a decline in the last third of the season. Furthermore, fitness coaches should incorporate considerations of playing at home and facing high-ranked opponents into their training periodization and match analysis, as these circumstances may elicit greater running demands compared to playing away or against lower-ranked opponents.
